# The 95% effective dose of nalbuphine in patient-controlled intravenous analgesia for patients undergoing laparoscopic total hysterectomy compared to equivalent sufentanil: Retraction

**DOI:** 10.1097/MD.0000000000029420

**Published:** 2022-06-17

The article, “The 95% effective dose of nalbuphine in patient-controlled intravenous analgesia for patients undergoing laparoscopic total hysterectomy compared to equivalent sufentanil”,^[1]^ which appears in Volume 99, Issue 22 of *Medicine*, is being retracted. The authors contacted the Editorial Office requesting a retraction due to an oversight in the clinical trial registration process and the recruitment of subjects before it was approved, which is not in accordance with formal clinical trial procedures. All authors agreed to retract this article.
